# Mutagenic Potentials of DNA Interstrand Cross-Links Induced by 7,8-Dihydro-8-Oxoadenine

**DOI:** 10.3390/molecules31020291

**Published:** 2026-01-14

**Authors:** Lillian F. Schmaltz, Nestor Rodriguez, Seongmin Lee

**Affiliations:** The Division of Chemical Biology and Medicinal Chemistry, College of Pharmacy, The University of Texas at Austin, Austin, TX 78712, USA

**Keywords:** 8-Oxoadenine, DNA interstrand cross-links, mutagenesis, oxidative damage

## Abstract

DNA interstrand cross-links (ICLs) are among the most cytotoxic forms of DNA damage, arising when the two strands of the DNA helix are covalently linked by crosslink-inducing agents such as bifunctional alkylating agents and reactive aldehydes. Several studies have demonstrated that ICLs can also be induced by reactive oxygen and nitrogen species. We previously reported that under oxidative conditions, the major oxidative adenine lesion 7,8-dihydro-8-oxoadenine (oxoA) can efficiently generate a novel class of oxoA-G ICLs, structurally resembling guanine–guanine (G–G) cross-links that can be induced by reactive nitrogen species. To investigate the mutagenic potential of these oxidation-induced ICLs in cells, we employed a *SupF*-based mutagenesis assay using bacterial cells. A single site-specific oxoA–G ICL was synthesized and incorporated into a plasmid, which was then introduced into an *E. coli* reporter strain to assess mutation profiles induced by both oxoA and oxoA–G ICLs. Our results show that oxoA–G ICLs cause A-to-C/T and G-to-C transversion mutations at the oxoA-G cross-link site, demonstrating highly promutagenic nature of the lesion in bacterial cells. We propose that the oxoA–G ICL may promote transversion mutations, likely driven by a syn conformer of unhooked oxoA-G ICL repair intermediates during translesion synthesis.

## 1. Introduction

**Figure 1:**
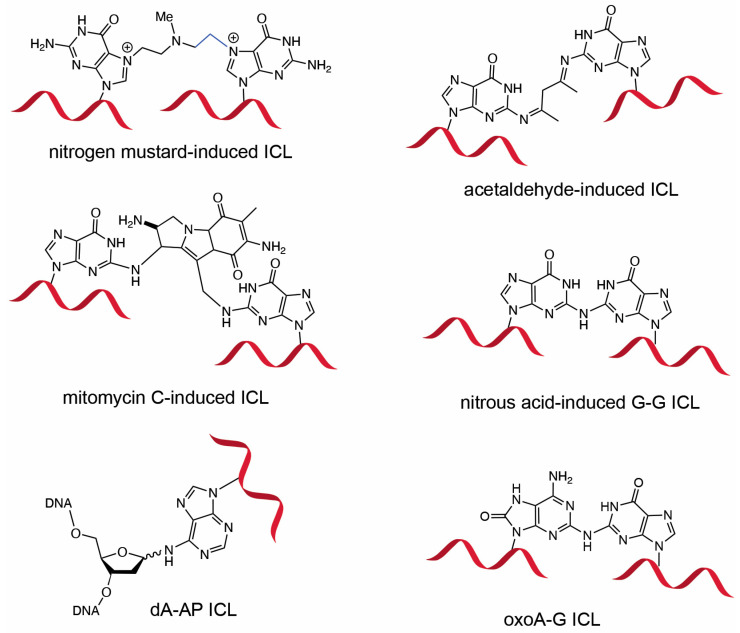
**DNA interstrand crosslinks induced by cross-linking agents**. Chemical structures of ICLs induced by nitrogen mustard, acetaldehyde, mitomycin C, nitrous acid, abasic site, and 8-oxoadenine are shown. The G-G ICL formed by nitrous acid is structurally similar to the oxoA-G ICL. Formation of the acetaldehyde-induced G-G ICL requires two molecules of acetaldehyde.

DNA interstrand cross-links (ICLs) are highly deleterious lesions formed when crosslink-inducing agents covalently modify opposing strands of the DNA duplex. If left unrepaired, ICLs prevent strand separation, thereby disrupting essential cellular processes such as transcription and replication, ultimately promoting genomic instability and carcinogenesis [1,2,3]. Due to their cytotoxicity, both synthetic and naturally occurring ICL-inducing agents—such as nitrogen mustards, psoralen, mitomycin C, cisplatin—have been employed in anticancer chemotherapy (Figure 1) [4,5,6,7]. In addition, the search for endogenous sources of ICLs has led to the identification of several novel classes of ICLs including those induced by alcohol, unsaturated aldehydes, and abasic sites [8,9,10]. In particular, it is well established that colibactin, a genotoxin produced by certain strains of *Escherichia coli*, can induce ICLs and has been implicated in the development of colon cancer, highlighting the pathological relevance of ICLs in microbe–host interaction and cancer etiology [11,12]. ICLs are also of particular relevance to the pathology of Fanconi anemia (FA), a rare genetic disorder caused by mutations in a network of genes responsible for replication-coupled ICL repair [13,14]. Interestingly, cell lines derived from patients with Fanconi anemia exhibit marked sensitivity to oxidative stress [15,16,17], suggesting that oxidative conditions may promote the formation of ICLs from oxidized lesions such as 8-oxopurines.

Oxidative damage to DNA is primarily caused by reactive oxygen species (ROS) [18,19], which are generated from both endogenous and exogenous sources, including cellular metabolism, tobacco smoke, aerobic respiration, ionizing radiation, and inflammatory responses [20,21,22]. In human cells, oxidative stress induces an estimated 10,000 DNA lesions per cell per day [23]. To maintain genomic integrity, cells counteract ROS through antioxidant systems such as glutathione and DNA repair mechanisms; however, excessive ROS levels lead to oxidative stress and DNA damage [24]. Within duplex DNA, purines are more susceptible to oxidation due to their lower redox potentials, resulting in the formation of 7,8-dihydro-8-oxoguanine (oxoG) and 7,8-dihydro-8-oxoadenine (oxoA) as major oxidative lesions [25]. While oxoG has been extensively studied and its mutagenic potential well characterized [26], oxoA has received comparatively less attention. Early studies indicated that oxoA is less mutagenic than oxoG in bacterial systems [27]; however, in mammalian cells, oxoA exhibits mutation frequencies comparable to oxoG [28,29]. Recent structural and kinetic data from our group have shown that bypass of oxoA by human TLS polymerase η is highly error-prone [30,31], underscoring the promutagenic nature of oxoA in mammalian cells.

**Figure 2:**
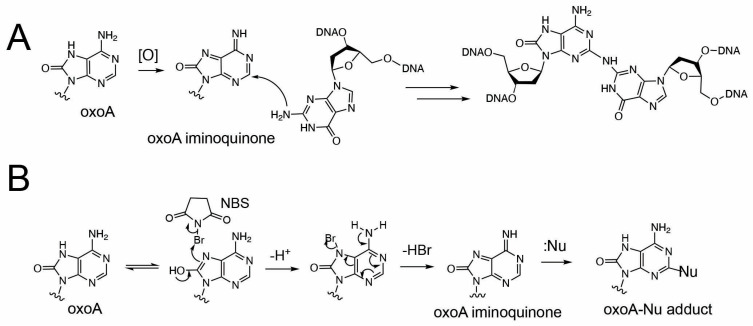
**Proposed formation of 8-oxoadenine-mediated ICL**. (**A**) Proposed cross-linking reaction between oxoA iminoquinone and guanine. The oxidative activation of oxoA would generate the highly electrophilic oxoA iminoquinone, which is attacked by neighboring guanine N2 to give the chemically stable oxoA-G cross-link. The covalent linkage between C2 of oxoA and N2 of guanine is shown. (**B**) Proposed production of oxoA-nucleophile cross-link via NBS-mediated oxidation of oxoA. Nu stands for nucleophile.

Oxidized bases possess significantly lower redox potentials than their unoxidized counterparts and are thus prone to further oxidation [25]. Under oxidative conditions, oxoA can undergo secondary oxidation to form cytotoxic ICLs with guanine (Figure 2). We have previously characterized the formation and structures of oxoA ICLs using various oxidative conditions using modified nucleobases. In particular, an MPO/H_2_O_2_/Cl^−^ system was used to mimic chronic inflammatory environments, which are associated with elevated cancer risk [32]. Under these conditions, oxoA–G ICLs formed with yields of approximately 16%, suggesting that oxoA-induced ICL formation could occur in vivo under oxidative stress. Mechanistic studies have shown that secondary oxidation of oxoA produces an oxoA iminoquinone intermediate, which can react with various nucleophiles at the C2 position of the iminoquinone [33]. Within dsDNA, the C2 of oxoA iminoquinone is susceptible to nucleophilic attack by the N2 of an opposing guanine, leading to the formation of the oxoA–G ICL [34]. Notably, oxoG does not form cross-links under similar conditions, highlighting the latent alkylation potential of oxoA and the need for further investigation. The structure of oxoA-G ICL is expected resemble that of G-G ICL induced by the endogenous signaling molecule nitric oxide (Figure 1), in which the C2 of guanine is covalently linked to the N2 of the opposing guanine within 5′-CG/GC-5′ sequences [3,35,36,37].

Cells, both healthy and cancerous, possess orchestrated mechanisms to repair or bypass ICLs during replication and transcription. However, ICL repair can be error-prone and may lead to oncogenic mutations, contributing to the development of secondary malignancies following chemotherapy [1,6,38,39]. The repair of ICLs is a complex, multi-pathway process involving nucleotide excision repair (NER), homologous recombination (HR), translesion synthesis (TLS), and the Fanconi anemia pathway [40]. These pathways collectively proceed through three major steps: lesion recognition, unhooking of the cross-link, and translesion bypass of the unhooked ICL repair intermediate. In the presence of a homologous template, ICL repair is typically error-free. However, in recombination-deficient cells, lesion bypass is primarily mediated by TLS [41,42]. Unhooking the ICL from either DNA strand generates a bulky DNA adduct, which must be bypassed by a TLS polymerase. This bypass step is inherently mutagenic due to the low fidelity of TLS polymerases [43,44]. Previous studies have shown that unhooked DNA fragments can range from 20 to 30 nucleotides in length [45]. The unhooking and TLS phases are particularly relevant to the mutagenic potential of oxoA and oxoA–G ICLs. Notably, the mechanisms of ICL recognition and bypass are influenced by both the structural features of the lesion and the cell cycle stage [46].

Given DNA’s vulnerability to oxidative stress and oxoA’s ability to form secondary lesions such as oxoA–G ICLs, our investigation aims to elucidate how these lesions contribute to genomic instability. Here, we report the mutagenic potential of oxoA and the oxoA–G ICL using a SupF-based mutagenesis assay [47,48,49,50,51]. Through site-specific incorporation of each lesion into plasmid DNA, we assessed their mutagenicity in bacterial cells. Our results demonstrate that the oxoA–G ICL is highly mutagenic and induces three distinct mutation types, which depend on the strand unhooked during ICL repair. This study provides new insights into oxoA-mediated mutagenesis and its role in genome instability.

## 2. Results and Discussion

**OxoA is nonmutagenic in bacterial cells**. To systematically study oxoA-mediated mutagenesis in bacteria, we transformed oxoA-containing pSP189 into the MBM7070 *E. coli* reporter strain. We also transformed unmodified pSP189 into MBM7070 to serve as both a positive transformation control and negative mutagenesis control. Over 1000 clones of unmodified pSP189 were observed. For oxoA-containing pSP189, we observed 546 blue colonies (Table 1). Random colonies were selected, amplified, and sequenced to confirm the absence of mutations at the lesion site. Sequencing also confirmed there were no issues with ligation. The mutagenic frequency of oxoA-containing pSP189 was identical to that of control pSP189, suggesting that oxoA is not mutagenic in bacteria. OxoA has largely remained overlooked because it was previously shown to be non-mutagenic in prokaryotes. In an early study using a site-specific oxoA lesion and *E. coli* DL7 cells, the mutation frequency for oxoA was calculated to be between 0.2 and 0.3% and almost identical to that of an adenine-containing control [27]. Our data supports that oxoA is not mutagenic in prokaryotes.

Table of mutation frequencies. Mutation frequencies were calculated as (white colonies/total colonies) × 100 and are reported as percent ± standard error of the mean (SEM). Total number of colonies analyzed for control, oxoA-containing, and oxoA-G ICL-containing pSP189 were >1000, 546, and 170, respectively.

OxoA is a relatively small lesion that does not perturb the DNA B-helix nor interfere with replication [52]. Oxidative damage, including oxoA, is repaired by base excision repair (BER) [53]. It has been shown that human thymine-DNA glycosylase (hTDG) and *E. coli* mismatch-specific uracil glycosylase (MUG) can efficiently remove oxoA:T, oxoA:C, and oxoA:G base pairs, which may explain why oxoA was not mutagenic in our *supF* mutagenesis assay [54,55]. Furthermore, oxoA does not block prokaryotic replicative polymerases. dTTP is readily inserted opposite templating oxoA [52,56]. While oxoA is not mutagenic in bacteria, other studies have shown that oxoA is mutagenic in mammalian cells [28].

**Figure 3:**
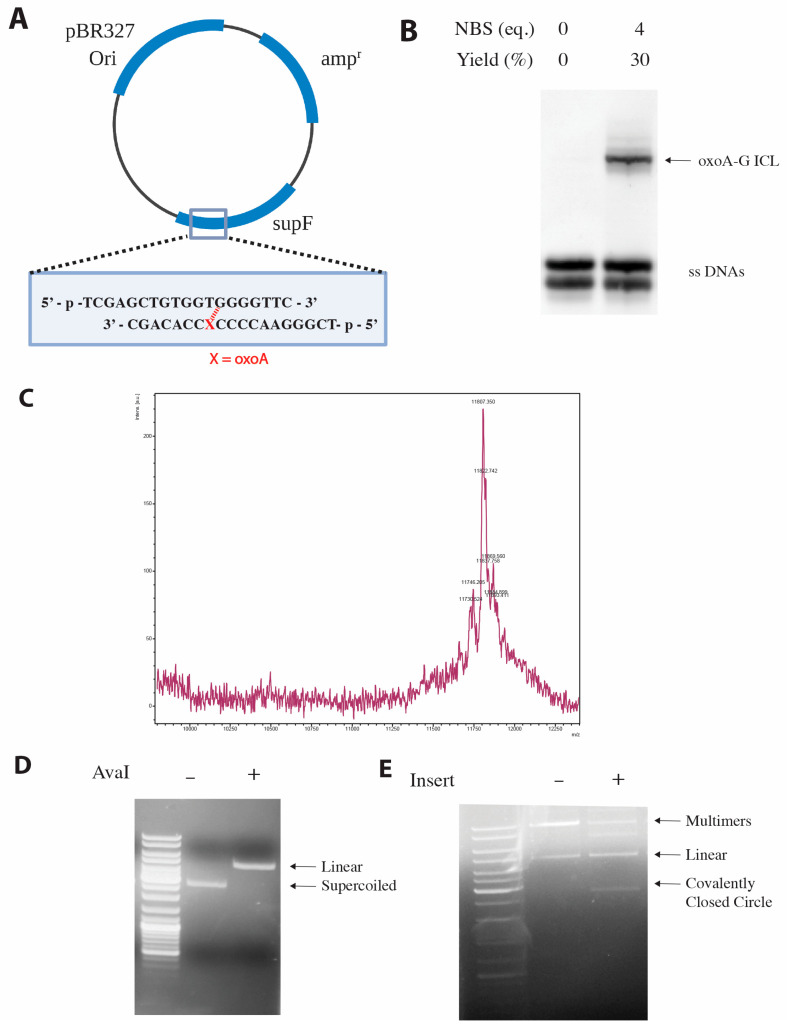
**Site-specific incorporation of oxoA and oxoA-G ICL into pSP189 vector**. (**A**) Plasmid map of the pSP189 vector. The oxoA lesion (denoted by X) is positioned at the fifth nucleotide of the *supF* gene. Inserts containing oxoA- or oxoA-G ICL were ligated into the *supF* gene. (**B**) Urea PAGE analysis of the cross-linking reaction with *N*-bromosuccinimide (NBS). The major oxoA-G ICL product was extracted, purified, and used as an insert to generate oxoA-G ICL-containing pSP189 plasmid. (**C**) MALDI-TOF mass spectrum of the oxoA-G ICL-containing insert, with an observed *m*/*z* of 11,807.8 (calculated *m*/*z* = 11,807.6). (**D**) Agarose gel electrophoresis following linearization of pSP189 with the AvaI restriction enzyme. (**E**) Agarose gel electrophoreses following ligation of the oxoA-G ICL-containing insert into pSP189. Supercoiled DNA bands were extracted, purified, and used in the *supF* mutation assay.

**Preparation of oxoA-G ICL-containing pSP189 plasmid**. OxoA has been shown to induce interstrand cross-links (ICLs) with guanine under oxidative conditions [34]. To evaluate if oxoA-induced ICLs may confer mutations, we first prepared oxoA-G ICL-containing DNA by treating the annealed, oxoA-containing insert with NBS (Figure 3B). Specifically, we inserted the oxoA lesion at the 5th nucleotide position within *supF*, a position where the oxoA may preferentially crosslink with the guanine flanking the 3′ of the base paired thymine. Oxidation-induced cross-linking mainly generated oxoA-G ICL product containing 5′-GTG-3′/3′-C(oxoA)C-5′ (30% yield, cross-linking sites are indicated underlined). The covalent linkages were inferred from previous studies demonstrating that oxoA preferentially links to the 3′-G of the opposing strand rather than the 5′-G [34]. We scaled up the reaction to cross-link 20 nmol of DNA, had its gel extracted, and purified the major and minor cross-linked products. The major cross-linked product was ligated into pSP189, and a negative control that did not include the double-stranded insert was run alongside the experimental reactions. We confirmed that the plasmid was not self-ligating and that supercoiled, ICL-containing plasmid was produced via agarose gel electrophoresis (Figure 3C,D).

**OxoA-G ICL is highly promutagenic in bacterial cells**. Although oxoA was not mutagenic in *E. coli*, we speculated whether the secondary oxoA-G ICL lesion would be. We transformed gel-extracted, unmodified pSP189 into *E. coli*, serving as a positive transformation control, alongside a negative mutagenesis control, and we observed over 1000 independent colonies. Alongside our controls, we transformed pSP189 containing the site-specific oxoA-G ICL into MBM7070 and observed 170 independent colonies (Table 1). Only blue colonies were observed with the control. Conversely, for the ICL-containing plasmid, we observed blue, white, and light-blue colonies. All white and light-blue colonies as well as a random sampling of blue colonies were grown up, and the plasmids were sequenced to evaluate the mutational spectrum and confirm there were no false negatives or positives. Sequencing confirmed a mutation frequency of 19 ± 2.2% (Table 1).

**OxoA-G ICL predominantly induces transversion mutations at the cross-linking site**. ICLs are highly cytotoxic DNA lesions that require complex repair and bypass mechanisms due to their covalent linkage of opposite strands within duplex DNA. The repair pathway can be simplified into three steps: recognition, unhooking, and bypass. After recognition of the ICL, endonucleases will cut on either side of the lesion to produce an unhooked intermediate. Bypass of this unhooked intermediate by TLS polymerases can be mutagenic [1,40,57].

**Figure 4:**
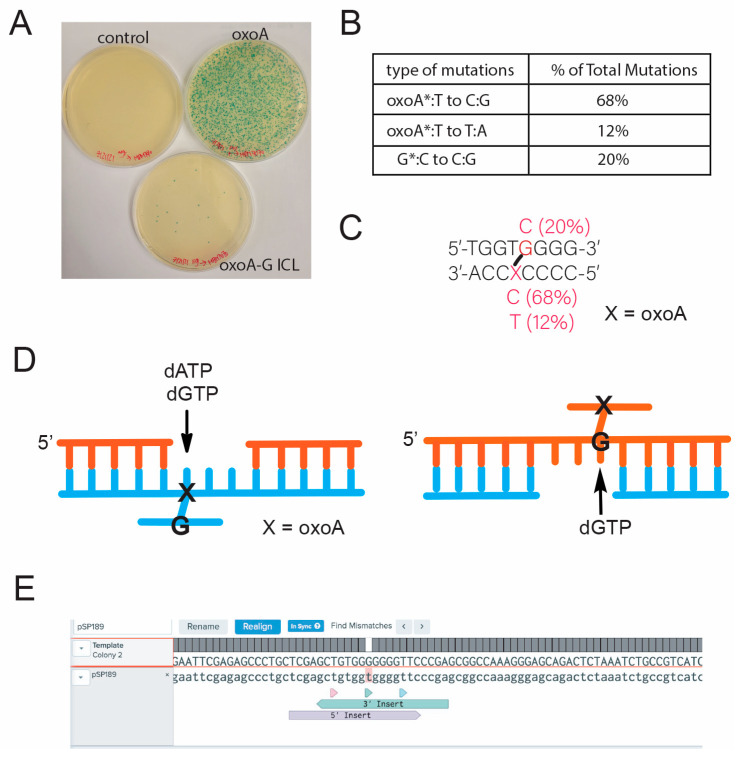
**Mutational spectrum of oxoA-G at the crosslinking site**. (**A**) Representative plate images from the blue-white colony screening of control-, oxoA-, and oxoA-G ICL-containing pSP189 plasmids. (**B**) Mutation spectrum induced by oxoA-G ICL. (**C**) Sequence map of the mutation sites in relation to the oxoA-G ICL. All the base substitution mutations occurred at the oxoA-G cross-link site. (**D**) Schematic showing nucleotide insertion opposite the unhooked oxoA-G ICL intermediate that will result in the varying mutations. Note that purine nucleotides would be preferentially incorporated opposite the unhooked oxoA-G ICL intermediate. (**E**) Representative sequencing read for oxoA-G ICL-containing plasmid.

To gain a better understanding of the mutagenic potential, we evaluated the mutation spectrum of the oxoA-G ICL-induced mutations (Figure 4). We sequenced all white and light-blue colonies formed after incorporation of oxoA-G ICL-containing DNA to evaluate the present mutations and to eliminate any false positives. We also sequenced a random sampling of blue colonies to determine whether there were any false negatives. We saw no correlation between colony color (white or light-blue) and the type of mutation produced. Approximately 162 unique base substitutions have been identified that will render *supF* inactive. It is possible that colony color is a result of sites independent of the oxoA-G ICL that have also been mutated and result in a specific colony color [58]. Strikingly, sequencing revealed oxoA-to-C transversion mutations as the major mutation (68%) (Figure 4A). This can be explained by an unhooking of the crosslinked guanine strand, followed by dGTP insertion opposite the crosslinked oxoA, henceforth referred to as oxoA* (Figure 4C). Unhooking in this manner also would give rise to oxoA-to-T mutations, where dATP is inserted opposite oxoA* (12%). Presumably, unhooking of the oxoA strand would result in dGTP being incorporated opposite the cross-linked G (G* hereafter) because we observed G-to-C mutations at the cross-linked G site (Figure 4B). Interestingly, 80% of mutations detected were the result of bypass across oxoA*. It is unclear if this is due to a profound strand bias during the unhooking step or if oxoA* is more mutagenic than G*.

**Figure 5:**
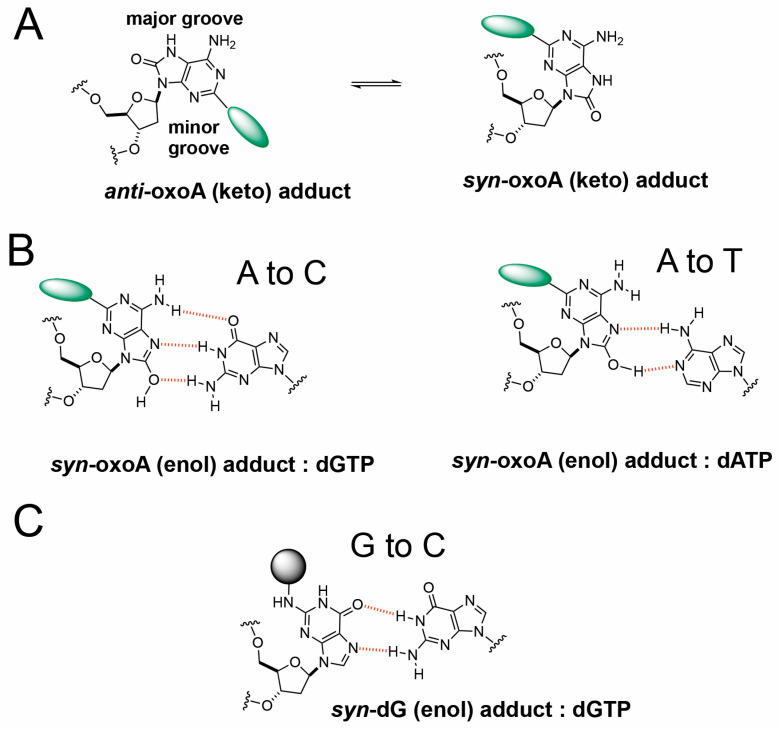
**Proposed mechanism of transversion mutations induced by unhooked oxoA-G ICL intermediate**. (**A**) *Anti* and *syn* conformers of oxoA adducts. The anti-conformer of oxoA adduct would experience steric clash with incoming nucleotides, facilitating the adoption of syn conformers during translesion synthesis. (**B**) Possible base pairings between syn-oxoA-dG adduct and dGTP or dATP during the bypass of unhooked oxoA-G ICL lesion, which would cause A-to-C and A-to-T transversions, respectively. Hydrogen bonds are shown as red dotted lines. (**C**) Possible base pairings between syn-dG-oxoA adduct and incoming dGTP during the translesion synthesis across unhooked oxoA-G ICL.

It is possible that oxoA* preferentially adopts a *syn* conformation due to the bulkiness and steric clash of the unhooked ICL adduct with surrounding bases and protein contacts (Figure 5A,B). Specifically, the covalent bond of the unhooked ICL would be at the C2 position of oxoA and extend from the Watson–Crick edge of oxoA, potentially interfering with insertion of an incoming nucleotide. A similar mechanism may explain the G-to-C transversions we observed. The unhooked ICL would produce a bulky lesion covalently bound to guanine (G*) at the N2 position (Figure 5C), a site involved in Watson–Crick hydrogen bonding. To accommodate this lesion, G may adopt a *syn* conformation and form a Hoogsteen base pair with an incoming guanine.

**OxoA-G ICL is significantly mutagenic in bacteria compared to other ICLs**. Our supF assay revealed the oxoA-G ICL produces a mutation frequency of 19% in bacteria, with oxoA-to-C being the predominant mutation analyzed. We propose that a *syn-anti* equilibrium exists to facilitate nucleotide misinsertion. Our mutation frequency is on par with that of mitomycin C, which forms an ICL between the N2 positions of opposing guanines: Zheng et al. showed that bypass of a site-specific mitomycin C ICL in a recombination-independent manner by DNA polymerase η (polη) resulted in a 22% mutation frequency in repair-proficient mammalian cells, while a cell line deficient in polη showed a drastic reduction in repair of the ICL. Similarly to our results, most mutations occurred from nucleotide insertion opposite one unhooked adduct [59]. When plasmids containing a site-specific mitomycin C crosslink were sequenced, three base substitutions were found: C-to-A transversions, C-to-T transitions, and C-to-G transversions. In contrast, our mutation frequency is significantly higher than that of other site-specific ICLs. A psoralen ICL resulted in an 8% mutation frequency when repaired in a recombination-independent pathway. Psoralen also intercalates the DNA helix but exhibits a different mutation spectrum. At the cross-linking site, there were both A-to-G transversions as well and T-to-G transversions [60]. For acetaldehyde-induced cross-links bypassed by dDNA polymerase κ, in both *E. coli* and human cells, the mutation frequencies were 3% and 4%, respectively [61]. In *xenopus* egg extracts, an acetaldehyde-ICL exhibited low mutation frequencies as well, at approximately 10% [9]. Repair of a site-specific cisplatin ICL induced a similar mutation frequency and was 97% error-free [62]. Additionally, cisplatin is a crosslinker that covalently binds the N7 positions of guanines [63]. Unhooking a cisplatin ICL would produce an adduct protruding from the N7 site on guanine, within the major groove of the helix. Thus, canonical Watson–Crick base pairing is possible, explaining the low mutation frequency for cisplatin ICLs. Compared with all the other site-specific cross-links, the mutation frequency of oxoA-G ICL is higher and underscores the promutagenicity of oxoA and oxoA-induced lesions.

**The crosslink structure may influence ICL-induced mutagenesis.** Varying pathways can be employed depending on the cell cycle stage and checkpoint at which the lesion is recognized [64]. For example, UHRF1 directly recognizes ICLs and is thought to recruit structure-specific endonucleases [65,66]. For eukaryotes, in G0 and G1 phases, ICL unhooking requires the NER pathway followed by TLS, whereas in the S phase, the HR pathway may be triggered by ICLs stalling replication forks and inducing double-strand breaks [57,67]. Additionally, the mode of DNA repair can be dependent on steric hindrance and the degree to which the DNA helix is distorted [57,68]. A nitrous oxide-induced crosslink between the N2’s of opposing guanine (e.g., PDB code: 1S9O) can provide insights into the structure of the oxoA-G ICL. The guanines are connected via a single NH group and the crosslink forms in the minor groove of the DNA helix [3]. A modeled structure of the oxoA-G ICL was compared to the structure of the G2-G2 ICL, and it is theorized that the structures would be very similar. Like the G2-G2 ICL, the oxoA-G ICL model features a widened minor groove near the crosslinking site, base flipping of the adjacent pyrimidines, and favorable stacking interactions [34]. Mitomycin C also sits in the minor groove, but due to its larger indoloquinone aromatic rings, the DNA helix is slightly perturbed [69]. Psoralens intercalate the DNA and cause helical unwinding [70]. The degree of helical distortion and sequence context may explain the different mutation frequencies of these site-specific ICLs.

Following recognition of the ICL, endonucleases unhook the DNA lesion. The structure of the unhooked intermediate may influence ICL-induced mutagenesis. Previous structural studies completed by our lab may provide insights into the mechanism of mutagenesis for oxoA-G ICL. Using a polβ host–guest complex system, Koag et al. solved the structures of templating oxoA within a single-nucleotide gapped DNA with and without an incoming dTTP and dGTP. The structures revealed that oxoA is conformationally flexible and exists in a *syn* and *anti* equilibrium. The *syn* conformations prevent steric clash between the oxo moiety of oxoA and the O4′ of the ribose sugar. When base pairing with an incoming dTTP, oxoA was in *anti* conformation and forms a Watson–Crick base pair; however, with an incoming dGTP, oxoA adopts a *syn* conformation and forms a Hoogsteen base pair with Watson–Crick-like geometry [30]. Furthermore, Jung et al. reported kinetic and structural data of *Sulfolobus solfataricus* DPO4 inserting a nucleotide opposite templating oxoA and highlighted the mutagenic potential of oxoA in bacteria. DPO4 inserted dTTP opposite oxoA with a relative efficiency of 0.55, suggesting that oxoA does not significantly alter correct insertion. Interestingly, oxoA significantly increased misinsertion by DPO4. DPO4 inserted dGTP opposite oxoA approximately 300 times more efficiently than a dA:dGTP insertion. Templating oxoA decreased the replication fidelity 560-fold, underscoring the mutagenic potential of oxoA in bacteria. To further highlight oxoA’s mutagenic potential in bacteria, the DPO4 structure of templating oxoA with an incoming dGTP was solved. OxoA adopts a *syn* conformation while dGTP is in *anti* conformation. OxoA:dGTP is able to form three Hoogsteen hydrogen bonds with Watson–Crick-like geometry [31].

While oxoA exists in a *syn-anti* equilibrium, a bulky adduct at the C2 position may push the balance towards the *syn* conformation. Unhooking of an ICL by an endonuclease will create a large intermediate. For example, the XPF/ERCC1 complex can cut DNA within 6 nucleotides of a crosslink. XPG, another nuclease implicated in NER, cuts the DNA on the 3′ side of the lesion. The dual incisions result in a fragment approximately 20 and 30 nucleotides long [64,71]. In an early study, researchers investigated unhooking of a psoralen ICL in cell extracts. Indeed, the unhooked oxoA intermediate, oxoA*, would include a bulky structure covalently bound to the C2 position of oxoA. The cross-linker would be a short, single-imino group and not as flexible as other ICLs such as the nitrogen mustards. To accommodate this unhooked oxoA-G ICL structure in the DNA helix and TLS polymerase active site, oxoA* may also adopt a *syn* conformation.

## 3. Materials and Methods

**Oligonucleotides used for ICL induction**. Modified oligonucleotides containing oxoA were custom synthesized by the Midland Certified Reagent Company (Midland, TX, USA). A 19-mer oligonucleotide (5′-phosphate-TCGGGAACCCC[oxoA]CCACAGC-3′) and its complementary strand (5′-phosphate-TCGAGCTGTGGTGGGGTTC-3′) were used for crosslinking reactions. The oligonucleotides were purified by urea PAGE and annealed in 10 mM phosphate buffer (pH 7) and 100 mM NaCl at 90 °C for 5 min and slowly cooled to room temperature. The annealed dsDNA has 5′ and 3′ sticky end overhangs that are complementary to the AvaI recognition sites.

**ICL induction with *N*-bromosuccinimide**. A stock solution of 5.8 mg/mL *N*-bromosuccinimide (NBS) was prepared from pure, recrystallized NBS. A tube of 20 nmol of DNA in 380 µL of annealing buffer (10 mM phosphate buffer (pH 7) and 100 mM NaCl) was incubated at 37 °C for 5 min. The cross-linking reaction was performed by adding 3 μL of NBS solution (95 nmol) every 15 min for a total of 1.25 h (25 molar equivalents of NBS) at 37 °C. The reaction mixture was subjected to a 3 kD MWCO centrifugal filter (Millipore Sigma, Burlington, MA, USA) to remove the oxidant NBS. Following the filtration, the reaction was dried on a SpeedVac (Eppendorf, Hamburg, Germany) and resuspended in 40 µL ultrapure water and 40 µL 2X formamide loading buffer (95% formamide, 5 mM EDTA, 0.025% (*w*/*v*) bromophenol blue). The reaction products were separated on an 18% Urea gel and the crosslinked products were gel-extracted and purified with a C18 Sep-Pak desalting column (Waters Corp., Milford, MA, USA). Confirmation of oxoA–G cross-linking was obtained by MALDI-TOF-MS analysis of the PAGE-purified band, which yielded an observed *m*/*z* of 11,807.8 (calculated *m*/*z* = 11,807.6), consistent with a cross-linked duplex containing an oxoA–G ICL formed via NBS-mediated oxidation.

**Construction of oxoA- and oxoA-G ICL-containing pSP189 plasmids**. The plasmid pSP189, which possesses the *supF* gene encoding a suppressor tRNA, was used as a vector for the oxoA ICL insert. There are two distinct restriction cut sites for the restriction enzyme, AvaI, within the *supF* gene. pSP189 was digested with AvaI at 37 °C overnight. The digest was visualized on a 1% agarose gel and purified with a Qiagen PCR Purification Kit (Germantown, MD, USA). To ligate, 20 μL reaction mixtures containing 0.04 pmol pSP189 and 0.12 pmol of ICL-containing insert were incubated with 1 μL T4 DNA Ligase at 16 °C overnight. Negative controls were also included that did not have insert in the reaction mixture. The ligation mixture was run on a 0.8% agarose gel, and it was confirmed that negative controls did not have self-ligation occurring. The reaction was scaled up to 500 μL to ensure the lesion-containing plasmid came from the same preparation. Supercoiled DNA bands were extracted and purified using the Qiagen Gel Extraction kit.

**Transformation into MBM7070**. The MBM7070 wildtype *E. coli* reporter strain (gift from Seidman lab, NIH, Bethesda, MD, USA) was used for blue-white colony screening. pSP189 containing the site-specific oxoA lesion or the oxoA-G ICL was electroporated into the cells and recovered at 37 °C in SOC. The cells were then plated on LB agar supplemented with 50 µg/mL ampicillin, 100 mM IPTG, and 40 µg/mL X-gal, and they were incubated at 37 °C overnight.

**Assessing mutation frequency and spectrum**. The mutation frequency was calculated as (white colonies/total colonies) × 100. All mutation frequencies were calculated for independent experiments and reported as the mean value ± standard error of the mean. All white colonies were sequenced to assess mutational spectrum and parse through false positives. A random sampling of blue colonies was sequenced for every plate to identify false negatives (UT Austin DNA Sequencing Facility, Austin, TX, USA). Colony sequencing results were aligned to the template ICL-containing plasmid sequence using the Benchling alignment software (https://www.benchling.com/alignments, accessed on 29 December 2025, Benchling, San Francisco, CA, USA).

**Figure 6:**
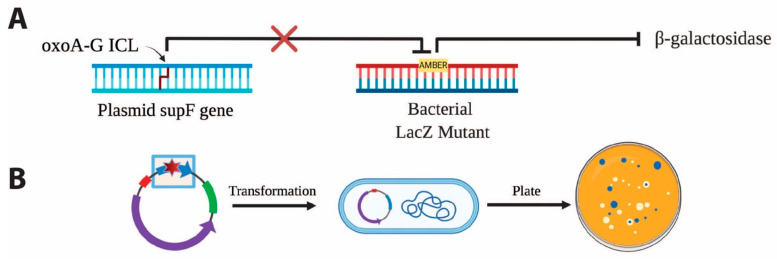
**Workflow of *supF*-based mutagenesis assay for oxoA-G ICL**. (**A**) The tyrosine amber suppressor gene, *supF*, suppresses the premature stop mutation in the *lacZ* gene of *E. coli* mutant MBM7070, resulting in the expression of functional B-galactosidase. Mutagenic repair of the oxoA-G ICL incorporated into the *supF* gene results in the persistence of the lacZ amber mutation, knocking out B-galactosidase activity. (**B**) Independent clones transformed with the modified plasmid and plated in X-gal containing media results in blue wildtype colonies and white mutagenic colonies. The oxoA or oxoA-G ICL is placed at the 5th nucleotide position within *supF*. Upon transformation into MBM7070, mutation frequency can be calculated by the blue-white colony screen in which mutation frequency = (white colonies/total colonies) × 100.

**Site-specific incorporation of oxoA and oxoA-G ICL in pSP189**. We previously reported the discovery of a new class of ICLs that arise when oxoA undergoes a secondary oxidation reaction and cross-links with an opposing guanine to form oxoA-G ICLs [34]. We employed the *supF* forward mutation assay to systematically evaluate the mutagenicity of oxoA and oxoA-G ICL in *E. coli*; the *supF* assay has been used extensively to evaluate the mutagenic potentials of DNA lesions including those induced by UV irradiation, nitric oxide, mitomycin C, and psoralen, and it enables visualization of mutagenesis via blue-white screening with ease (Figure 6) [48,49,50,51,52]. To investigate the mutagenic potentials of both oxoA and its secondary lesion, oxoA-G ICL, we incorporated both lesions within the *supF* gene on pSP189 at a specified site. This vector includes the pBR327 ori sequence that enables plasmid replication within *E. coli*. There are two *AvaI* recognition sites within *supF* that were used to incorporate the lesions at the 5th nucleotide position of *supF*. We monitored successful ligation via agarose gel electrophoresis. A negative control reaction was prepared alongside our experimental reaction which did not include the double stranded DNA insert. For this negative control, no supercoiled plasmid was observed, indicating that our linearized vector was not self-ligating without insert. Upon observation of the oxoA-containing supercoiled plasmid, the plasmid product was isolated by gel extraction.

## 4. Conclusions

Our studies demonstrate that whereas oxoA itself is non-mutagenic in bacterial cells, oxoA–G ICLs are highly promutagenic, exhibiting a mutation frequency of 19%—comparable to that of mitomycin C and substantially higher than other site-specific ICLs such as psoralen, acetaldehyde, and cisplatin. The predominant A-to-C/T transversions suggest that mutagenic bypass of the unhooked oxoA lesion (oxoA*) is a major contributor to the observed mutations. The bulky ICL adduct likely imposes structural constraints that promote syn conformations of the templating base of the unhooked oxoA-G adduct and non-canonical base pairing, thereby facilitating misincorporation during translesion synthesis. These findings underscore the strongly promutagenic nature of oxoA-induced ICLs and highlight the critical influence of lesion structure and repair pathway dynamics on mutational outcomes. Given the ubiquity of oxidative stress in biological systems, oxoA–G ICLs may represent a previously underappreciated source of genomic instability.

## Figures and Tables

**Table 1:** Mutation frequencies of oxoA- and oxoA-G ICL-induced mutations.

DNA	Mutation Frequency (%)
Control	<0.1
oxoA-containing pSP189	<0.1
oxoA-G ICL-containing pSP189	19 ± 2.2

## Data Availability

All supporting data are included within the main article. Original data including gel images (Figure 3) will be available upon request.
